# The mechanism of m6A methylation analysis of the transcriptome to regulate the diameter of Alpine Merino wool fiber

**DOI:** 10.5713/ab.25.0347

**Published:** 2025-08-25

**Authors:** Lin Yue, Tingting Guo, Bowen Chen, Jianbin Liu, Zengkui Lu, Chao Yuan

**Affiliations:** 1 Lanzhou Institute of Husbandry and Pharmaceutical Sciences, Chinese Academy of Agricultural Sciences, Lanzhou, China; 2 Sheep Breeding Engineering Technology Research Center of Chinese Academy of Agricultural Sciences, Lanzhou, China; 3 Key Laboratory of Animal Genetics and Breeding on Tibetan Plateau, Ministry of Agriculture and Rural Affairs, Lanzhou, China

**Keywords:** Alpine Merino Sheep, lncRNA and circRNA, m6A Methylation, Wool Fiber Diameter

## Abstract

**Objective:**

Wool serves as an important textile raw material, with fiber diameter being a major determinant influencing the economic value and quality of wool products. Investigating the regulatory mechanisms that influence wool fiber diameter is necessary for formulating aimed at improving wool fineness. We used methylationomics to examine the skin tissue of individuals with different fiber diameters, and investigated the regulation mechanisms influencing wool fiber diameter.

**Methods:**

In this study, we analyzed the transcriptome and m6A methylome of skin tissues from individual Alpine Merino sheep, classified into three groups based on wool fiber diameters, to identify key methylated RNAs and explore the role of m6A methylation in regulating this trait.

**Results:**

A total of 54,057 methylated peaks, 4,273 differentially methylated genes, 139 differentially methylated lncRNAs, and 2,992 differentially methylated circRNAs were found in the three comparisons. These gene loci showed enrichment in the Wnt, Notch, and TGF-β signaling pathways, as determined through Gene Ontology and Kyoto Encyclopedia of Genes and Genomes pathway analyses. RNA correlation analyses revealed key RNAs, such as *CACNA1E*, *FOS*, *CAMK2B*, *RNF43*, circ-0317, circ-4794, TCONS-00020832, and TCONS-00020845, indicating that hypermethylation may play an important role in affecting wool fiber diameter.

**Conclusion:**

The findings elucidate the molecular regulatory mechanisms underlying wool fiber diameter and provide a theoretical foundation for advancements in the wool industry.

## INTRODUCTION

Alpine Merino sheep is a new breed for wool and meat production adapted to cold and arid ecological zones in the high mountains. The species has a wool fiber diameter of 19.1–21.5 μm, providing excellent germplasm resources for producing high-quality wool [1]. Wool is the first natural fiber in the history of textile use and an important raw material in the modern textile industry. Fiber diameter is an important factor determining the economic value of wool, and high-end wool products have stringent wool fineness requirements; in particular, ultra-fine wool is most popular. Therefore, the study of biological mechanisms regulating the diameter of wool fibers is of great significance for the development of the wool industry. Valuable information on the molecular basis of fiber diameter has been obtained through omics methods. For example, Zhang et al [2] identified KRTAP15-1, KRTAP3-1, and other proteins related to wool fiber diameter through proteomics. Yue et al combined transcriptome and metabolomics analyses to reveal key genes and metabolites related to wool fiber diameter [3]. Pu et al [4] analyzed the hair follicle transcriptomes of six sheep breeds, and discovered candidate genes, *KAZALD1*, *MYOC*, and *C1QTNF6*, involved in the hair follicle cycle and regulation of wool fineness.

m6A methylation is a common form of chemical modification on RNA; in this process, the carbon atom at the N6 position on an adenine nucleotide in an RNA molecule is replaced with a methyl group. This modification affects RNA translation, shear, and degradation and regulates a wide range of cellular processes [5,6]. Hua et al [7] investigated the differences between coarse and fine wool traits in Merino lambs based on m6A methylation, revealing key genes, such as DAR, FGF5, TCHH, and KRT2, and emphasizing the important role of the PI3K/AKT signaling pathway in the generation of the coarse wool phenotype. Using MeDIP-seq technology to study the methylation profiles of Merino sheep hair follicles at six time points in combination with transcriptome analyses, Tian et al [8] screened key genes enriched in the Wnt, TNF, and TGF-β pathways and showed that the WNT2 gene promotes skin hair follicle growth. During the growth and development of wool fibers in Liaoning Cashmere goats, m6A methylation-modified genes are mainly enriched in intermediate filaments and other filaments [9]. In addition, m6A methylation-modified circRNAs, such as circRNA-ZNF638, circRNA-STAM2, circRNA-TULP4, and circRNA-CAAP1, have regulatory roles in secondary hair follicle growth and development, and potentially affecting wool fiber diameter [10].

With the development of biotechnology, omics has become an important means to study the regulation of wool fiber diameter. In this study, we evaluated skin samples from individual Alpine Merino sheep classified into groups according to fiber diameter and searched for differentially methylated genes. Our results clarify the role of m6A methylation in the regulation of wool fineness and further elucidate the mechanism underlying variation in wool fiber diameter, providing a theoretical basis for the development of high-quality products in the textile industry.

## MATERIALS AND METHODS

### Experimental animals and traits

The Alpine Merino sheep used in this experiment originated from the Gansu Sheep Breeding and Promotion Station. All experimental animals were adult ewes from the same population. According to the coefficient of variation (CV) and mean fiber diameter (MFD), animals were divided into an SF group (MFD = 17.68±0.26 μm, CV<20%), EF group (MFD = 19.15± 0.37 μm, CV<22%), and M group (MFD = 22.51±0.43 μm, CV<23.6%). Three comparisons were set up according to the gradient of wool fiber diameters (EF/SF, M/EF, and M/SF). Three sheep were collected from each group, and the skin tissue was derived from the left shoulder of the experimental animal. The collection area was approximately 2 cm×3 cm. Samples were placed in a freezing tube, immediately frozen in liquid nitrogen, and stored at −80°C.

### RNA extraction and sequencing

Total RNA was extracted using TRIzol reagent (Invitrogen) according to the manufacturer’s protocol. RNA purity and quantification were evaluated using the NanoDrop 2000 spectrophotometer (Thermo Fisher Scientific). RNA integrity was assessed using the Agilent 2100 Bioanalyzer (Agilent Technologies). Libraries were constructed using the VAHTS Universal V6 RNA-seq Library Prep Kit according to the manufacturer’s instructions. The libraries were sequenced on the llumina NovaSeq 6000 platform and 150 bp paired-end reads were generated.

### m6A enrichment and RIP Library Construction

RNA Fragmentation Reagent (Invitrogen) was added to the RNA of skin tissue samples. The RNA was fragmented at 94°C for 5 min in a thermocycler. m6A-Dynabeads were added to bind to the fragmented RNA and rotated at 7 rpm for 1 h. m6A-Dynabeads-RNA complexes were resuspended in 500 μL of m6A Binding Buffer and incubated for 3 min at room temperature. The clear supernatant was removed after placing the beads in the magnet. Low Salt Buffer, High Salt Buffer, and TET buffer were added and the above steps were repeated. Subsequently, m6A-positive RNA was eluted, and the supernatant containing the eluted m6A RNA was collected in a new test tube and purified using phenol, chloroform, and ethanol. After drying, samples were suspended in Ultra-Pure H20.

Fragment, Prime, Finish Mix was added to the m6A-positive fragmented RNA. Samples were centrifuged at 4°C at 10,000×g for 7.5 min. Fragment sizes of each individual library were verified using Agilent BioAnalyzer 2100 or a High Sensitivity DNA chip. The library was quantified by quantitative polymerase chain reaction (qPCR) using the Kapa Library Quantification Kit according to the manufacturer’s instructions. The libraries were used for high-throughput sequencing.

### Data processing

Raw data in fastq format were first processed using Fastp [11]. HISAT2 [12] software was used to align clean reads to the reference genome of sheep (Oar _ v4.0). Using MeTDiff [13] software, input samples were used as controls for peak detection, setting FRAGMENT _ LENGTH = 200. Differential peak detection was performed using MeTDiff software, with screening conditions of p<0.05, fold change (FC)>1.5, and annotation of detected peaks. The R package Guitar [14] was used for statistical analyses and visualization of the distribution of peaks on gene bodies. MEME-CHIP and HOMER were used to detect sequence motifs. MEME-ChIP uses two programs, MEME [15] and Dreme [16], to detect significant motif sequences in the peak sequence. Homer software can perform motif analyses on peaks de novo and based on databases. Gene Ontology (GO) [17], and Kyoto Encyclopedia of Genes and Genomes (KEGG) [18] pathway enrichment analyses of peaks and differential peaks were performed using R based on the hypergeometric distribution.

FPKM [19] values for each gene were calculated and read counts of each gene were obtained using HTSeq-count [20]. DESeq2 [21] software was used to standardize the counts for each sample gene (using the BaseMean value to estimate the expression level), and the FC was calculated. The differentially expressed genes were screened according to the difference multiple and the difference significance test results. p<0.05 and |FC|>2 was set as the threshold for significantly differential expression gene (DEGs). GO, KEGG pathway enrichment analysis of DEGs were performed to screen the significant enriched term.

### Potential peak analysis of lncRNA and circRNA

The location information for the identified peaks was compared with known annotation information for lncRNAs and circNRAs in the NCBI and Ensembl databases. If there was overlap between the two, the peak might be on a lncRNA or circNRA. The lncRNA and circNRA counts for each sample were standardized using DESeq software (using BaseMean values), FC values were calculated, and the significance of differences in read counts was tested using the NB (Negative Binomial Distribution Test). Significantly differentially expressed lncRNAs and circNRAs (DELs and DECs) were screened according to p<0.05, |FC|>1.5. GO and KEGG pathway enrichment analyses of DEGs were performed to evaluate functional enrichment. The correlation coefficient (cc) and p-value for relationships between DEL, DEC, and differentially methylated genes (DMG) were analyzed using the R language cor function, and DELs, DECs, and DMGs with p<0.05 and |cc|>0.8 were screened to construct a regulatory network.

### Quantitative real time polymerase chain reaction validation

Four RNAs were randomly selected for real time polymerase chain reaction (RT-PCR) analysis, with the ARPC5 gene as an internal reference. The reactions were performed using the PerfectStart Green qPCR SuperMix kit on the LightCycler 480 II RT-PCR System (Roche). The expression levels of the genes were analyzed using the 2-ΔΔCt method. The relationship between the expression levels of these four genes in different groups and the sequencing data was analyzed using the cor function in R language.

## RESULTS

### Analysis of m6A methylation profiles

**Figure 1:**
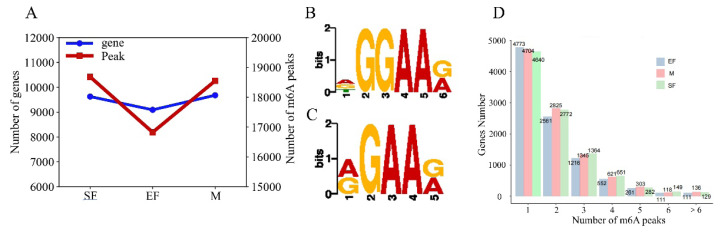
m6A peak analysis. (A) Comparison of the numbers of m6A peaks and m6A-modified genes. (B) Representative motifs of the SF group identified using Dreme. (C) Public motifs of the EF group and M group identified using Dreme. (D) Distribution of m6A peaks on genes.

**Figure 2:**
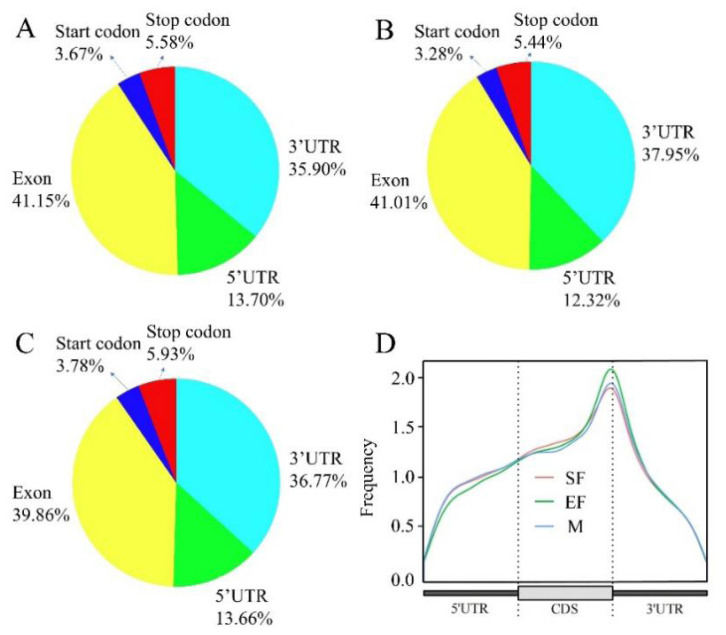
m6A peak gene functional element and location distributions. (A) m6A peak gene functional element distribution in the SF group. (B) Peak gene functional element distribution in the EF group. (C) Peak gene functional element distribution in the M group. (D) m6A peak location distribution.

Peak detection was performed on a genome-wide scale using MeTDiff software. The most peaks were detected in skin samples from the SF group, and the fewest peaks were detected in samples from the EF group; the number of peak modifier genes in the three groups was proportional to the number of detected peaks, with the fewest peak modifier genes in the EF group and similar numbers in the SF and M groups (Figure 1A). The total lengths of peaks detected in the SF, EF, and M groups were 10340817, 8732466, and 10107694 bp, with genomic shares of 40%, 33%, and 39%, respectively (Supplement 1). By analyzing significant peaks, we identified AGGAAG as a representative motif in the SF group and AGAAG in the EF and M groups; these sequences are frequently present in m6A peaks (RGAAR, R = A or G) (Figures 1B, 1C) [22]. Most genes in the SF, EF, and M groups contained only one m6A peak (Figure 1D), and these m6A peaks were mostly distributed in exonic regions of genes, followed by 3′-UTRs (Figures 2A–2C). The m6A sites were enriched near the 3′ end of the mRNA (Figure 2D).

### MRNA differential m6A peak analysis

**Figure 3:**
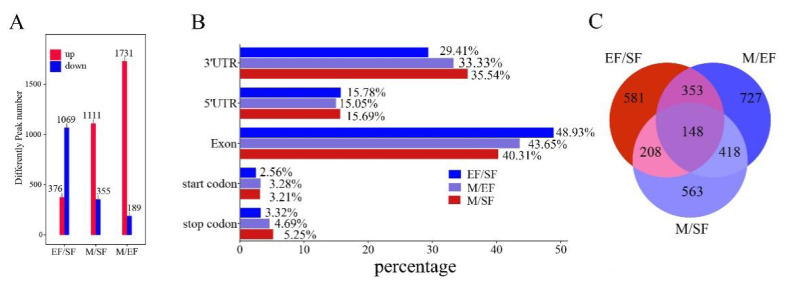
Differential m6A peaks in different comparisons. (A) Significantly different m6A peaks. (B) Venn diagram of significantly different m6A peaks. (C) Distribution of functional elements of differential m6A peak genes.

**Figure 4:**
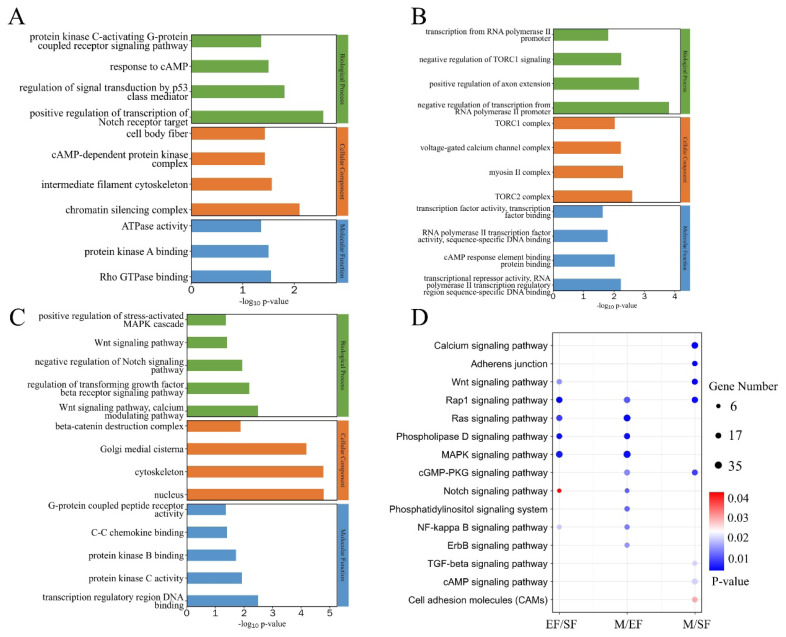
GO and KEGG analyses of genes in differential peaks. (A) GO analysis of differential genes in the EF/SF group. (B) GO analysis of differential genes in the M/EF group. (C) GO analysis of differential genes in the M/SF group. (D) KEGG analysis of differential genes. GO, Gene Ontology; KEGG, Kyoto Encyclopedia of Genes and Genomes.

After screening, the highest number of DMGs was obtained in the M/EF comparison, with 1,731 genes with significantly high methylation levels and 189 genes with significantly low methylation levels. The fewest differentially methylated genes were detected in the EF/SF comparison; different from the other comparisons, EF/SF showed far more DMGs with low methylation levels than with high methylation levels (Figure 3A). Most differential m6A peaks were distributed in exonic and 3′-UTR regions, consistent with the distribution of the detected m6A peaks (Figure 3B). A total of 148 DMGs common to the EF/SF, M/EF, and M/SF comparisons were obtained, as visualized using a Venn diagram analysis (Figure 3C). Subsequently, we analyzed the functions of DMGs in these three comparisons using GO and KEGG (Figure 4). DMGs in the EF/SF group were enriched in ATPase activity, intermediate filament cytoskeleton, regulation of signal transduction by p53 class mediator, positive regulation of transcription of notch receptor target, and other entries; these terms were mainly related to cell proliferation and differentiation and structure formation. Most DMGs in the M/EF group were enriched in negative regulation of transcription from RNA polymerase II promoter, transcription from RNA polymerase II promoter, transcriptional repressor activity, and RNA polymerase II transcription regulatory region sequence-specific DNA binding. These pathways regulate gene transcription and are involved in the ability of cells to respond to changes in the external environment. DMGs in the M/SF group were enriched in various functions, including the Wnt signaling pathway, negative regulation of Notch signaling pathway, and regulation of transforming growth factor beta receptor signaling pathway, which are closely associated with hair follicle development and hair coat phenotypes [23–26], as well as transcription regulatory region DNA binding, protein kinase B binding, protein kinase C activity, and other items related to gene expression regulation and cell signal transduction.

A KEGG analysis showed that DMGs in EF/SF, M/EF, and M/SF comparisons were enriched for pathways associated with follicle development and hair growth. DMGs in the EF/SF group were involved in the MAPK signaling pathway, NF-kappaB signaling pathway, Wnt signaling pathway, and Notch signaling pathway. DMGs in the M/EF group were involved in the MAPK signaling pathway, NF-kappaB signaling pathway, and Notch signaling pathway. DMGs were enriched in the Wnt signaling pathway and TGF-beta signaling pathway in the M/EF group.

### Correlation analysis of differential expression genes and differential m6A peaks

**Figure 5:**
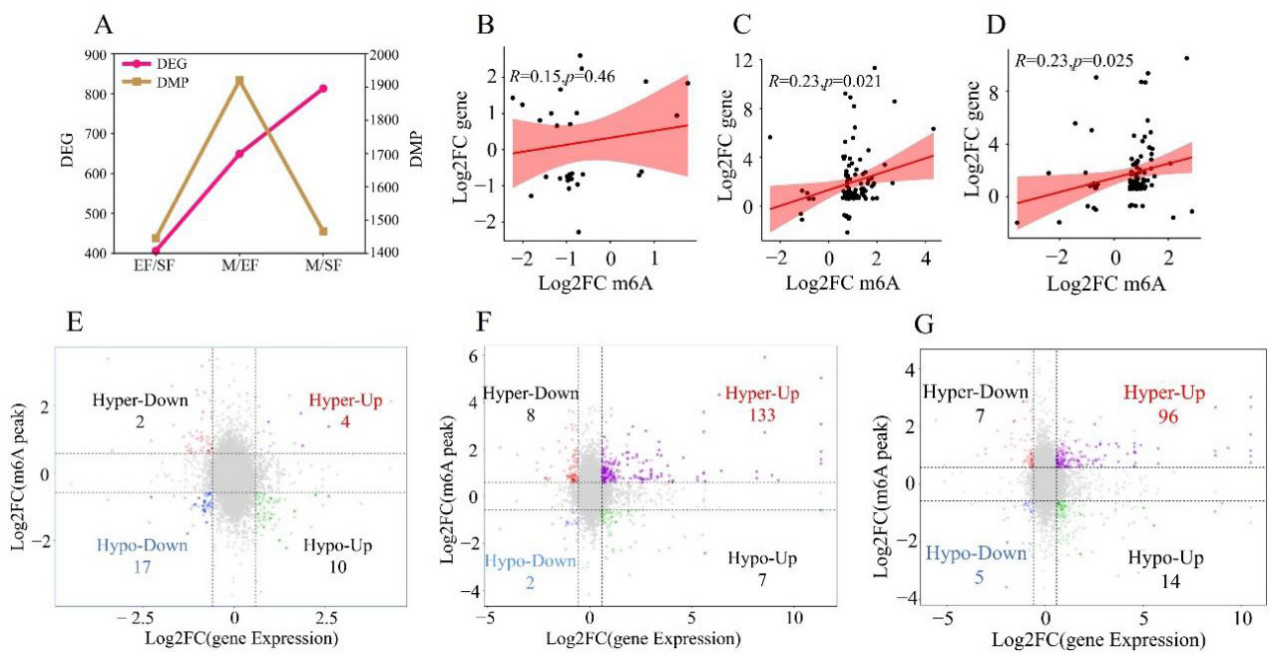
Joint analysis of DEGs and DMGs. (A) Numbers of DEGs and DMPs in different comparisons. (B) Correlation between DEGs and DMPs in the EF/SF group. (C) Correlation between DEGs and DMGs in the M/EF group. (D) Correlation between DEGs and DMGs in the M/SF group. (E) DEG and DMG joint analysis for the EF/SF group. (F) DEG and DMG joint analysis for the M/EF group. (G) DEG and DMG joint analysis for the M/SF group. DEG, differential expression gene; DMP, differentially methylated genes; FC, fold change; DMG, differentially methylated genes.

m6A methylation modification directly affects gene expression. We further analyzed the relationship between DMPs and DEGs. The differences among groups (i.e., EF/SF, M/EF, and M/SF) in the numbers of DMPs and DEGs were not consistent. The number of DEGs increased from EF/SF to M/EF and M/SF, while the number of DMPs was highest for M/EF (Figure 5A). These findings indicated that in the M/SF group, the primary cause of gene expression differences may not be m6A methylation. DMPs and DEGs were positively correlated in all three groups; however, the overall correlation was not strong (Figures 5B, 5C). A four-quadrant plot analysis showed 14 significantly up-regulated DEGs in the EF/SF group, including 4 hypermethylated and 10 hypomethylated genes, and 19 significantly down-regulated DEGs, including 2 hypermethylated and 17 hypomethylated. There were 140 significantly up-regulated DEGs in the M/EF group. Of these, 133 were hypermethylated and 7 were hypomethylated. There were also 10 significantly down-regulated DEGs. Of these, 8 were hypermethylated and 2 were hypomethylated. The M/SF group showed 110 significantly up-regulated DEGs, including 96 hypermethylated and 14 hypomethylated genes. Additionally, 12 significantly down-regulated DEGs were identified, of which 7 were hypermethylated and 5 were hypomethylated.

### Analysis of lncRNA and cirRNA potential m6A peaks

**Figure 6:**
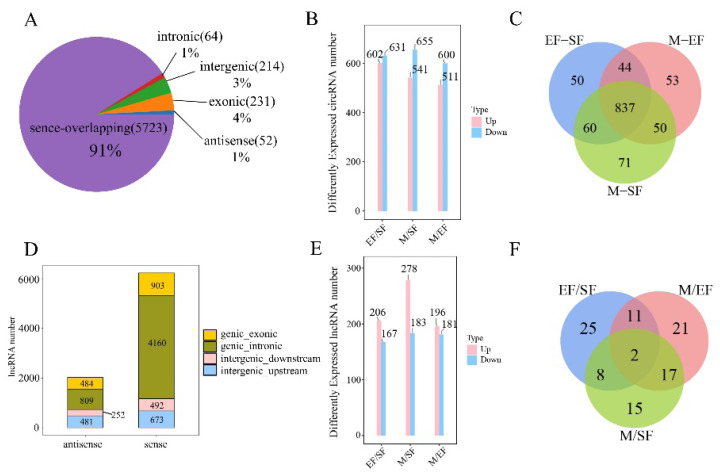
Identification and analysis of cirRNA and lncRNA peaks in skin tissues. (A) Classification of circRNAs. (B) Differential expression of circRNAs in three comparisons. (C) m6A peak Venn diagram of circRNAs. (D) Classification of lncRNAs. (E) Differential expression of lncRNAs in three comparisons. (F) lncRNAs on m6A peak Venn diagram.

Analysis of the sequencing data revealed 6284 circRNAs in skin tissues (Supplement 2), and 91% of the circRNAs were located in the sense-overlapping region (Figure 6A). There were 1,233 significantly differentially expressed circRNAs in the EF/SF group, 602 of which were significantly upregulated; 1,196 significantly differentially expressed circRNAs in the M/SF group including 541 that were significantly upregulated; and 1,111 significantly differentially expressed circRNAs in the M/EF group including 511 that were significantly upregulated (Figure 6B). These three comparisons shared 837 common DMCs. There were 8,823 lncRNAs (Supplement 3), the vast majority of which were sense lncRNAs (Figure 6D). In the EF/SF, M/SF, and M/EF groups, there were significantly more up-regulated DELs than down-regulated DELs. The M/SF group had the most significantly up-regulated lncRNAs with 278, the M/EF group had the fewest with 196, and the M/SF group had the fewest significantly down-regulated lncRNAs with 167 (Figure 6E). A Venn diagram analysis showed that there were two common DMLs in the three groups, of which TCONS_00020375 was significantly up-regulated in the EF/SF group and down-regulated in the M/EF group.

**Figure 7:**
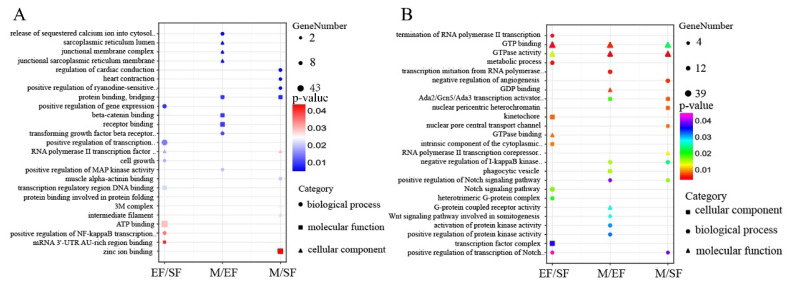
GO analysis of lncRNA versus cirRNA differential peaks. (A) lncRNA differential peaks. (B) cirRNA differential peaks. GO, Gene Ontology.

To analyze the functions of DECs and DELs, we performed GO and KEGG enrichment analyses. Most terms in the GO enrichment analysis for DECs and DELs in the EF/SF group were related to transcription and energy metabolism, such as termination of RNA polymerase II transcription, GTPase activity, positive regulation of transcription from RNA polymerase II promoter, as well as positive regulation of NF-kappaB transcription factor activity, Notch signaling pathway, and other critical pathways. DECs and DELs in the M/EF group were enriched in beta-catenin binding, negative regulation of I-kappaB kinase/NF-kappaB signaling, and positive regulation of Notch signaling pathway. DELs in the M/SF group were mainly enriched n cardiac function, muscle contraction and cell signaling. DECs were enriched in the positive regulation of Notch signaling pathway, negative regulation of I-kappaB kinase/NF-kappaB signaling, positive regulation of transcription of Notch receptor target, and other pathways related to hair follicles (Figures 7A, 7B).

A KEGG enrichment analysis provided additional information about the functions of DECs and DELs. DECs and DELs in the EF/SF group were mainly involved in pathways related to cell development, metabolic regulation, and immune response, such as Signaling pathways regulating pluripotency of stem cells, fat digestion and absorption, and Intestinal immune network for IgA production. In the M/EF group, DECs were enriched in classical pathways, such as the MAPK signaling pathway and PI3K-Akt signaling pathway, and DELs were enriched in pathways related to cellular metabolism, signaling regulation, and immune response. DECs were enriched in classical pathways, such as the MAPK signaling pathway and PI3K-Akt signaling pathway, and DELs were enriched n pathways related to cellular metabolism, signaling, and regulation of pathological states. Enriched pathways for DECs and DELs in the M/SF group were similar to those for the EF/SF group and mainly contributed to cellular metabolism, development, immune response, and disease genesis (Supplement 4).

**Figure 8:**
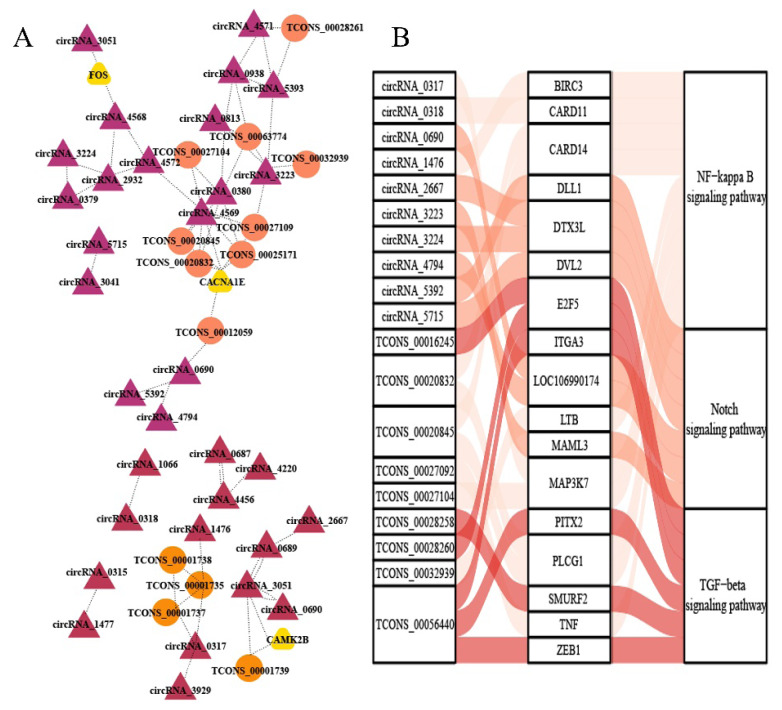
RNA interaction network. (A) Interaction diagram of key DMGs, lncRNAs, and circRNAs. (B) Sankey diagrams of DMGs, lncRNAs, and circRNAs. DMG, differentially methylated genes.

To further explore the relationships between lncRNAs and circRNAs (important factors affecting gene expression) an DMGs and DEGs, we mined the genes *FOS*, *CACNA1E*, and *CAMK28*, which were identified as both DMGs and DEGs and enriched in the follicle development-related pathway (Figure 8). By constructing a network diagram, we found that FOS was positively correlated with circRNA3051 and circRNA4568, which were positively correlated with the vast majority of other circRNAs and lncRNAs. *CACNA1E* was closely related to numerous circRNAs and lncRNAs, and most of these were positively correlated. *CAMK28* was mainly associated with TCONS_00001739, circRNA_3051, and circRNA_3051, with positive correlations.

### Validation of expression levels by real time polymerase chain reaction

**Figure 9:**
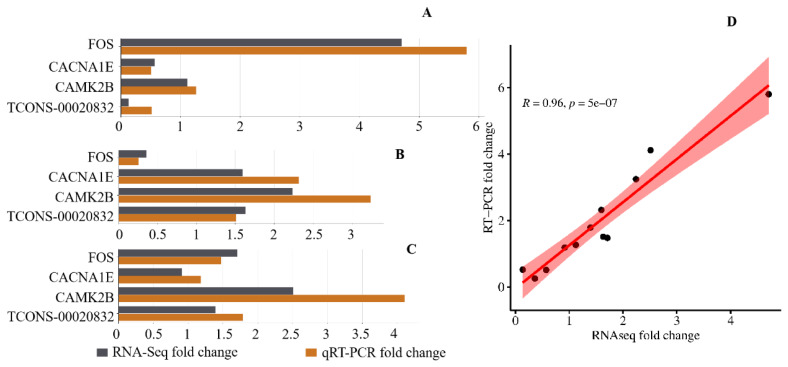
qRT-PCR validation of differentially expressed genes. (A) qRT-PCR expression levels in the EFSF group. (B) qRT-PCR expression levels in the MEF group. (C) qRT-PCR expression levels in the MSF group. (D) Correlation between qRT-PCR and RNA-Seq mRNA expression levels. qRT-PCR, quantitative real time polymerase chain reaction.

We selected four RNAs, namely *FOS*, *CACNA1E*, *CAMK2B*, and TCONS-00020832, and analyzed their expression levels across different groups with varying fiber diameters. The expression levels measured by qRT-PCR showed an extremely strong correlation with the RNA-Seq data (Figure 9D), thereby confirming the authenticity and reliability of the RNA-Seq data in this study.

## DISCUSSION

Wool is an important raw material for the textile industry, and its fineness affects the economic value and quality of wool products. Wool fineness is affected by age, variety, nutrition, and other factors [27–29], including many interacting biological mechanisms. Previous studies on the regulation of wool fiber diameter have focused on a single gene or protein, and omics analyses have focused on the transcriptome and proteome [30,31]. These analyses have yielded many valuable results regarding wool fiber diameter regulation. However, mRNA- and protein-level analyses alone cannot capture the complete biological regulatory mechanism. Some scholars have analyzed the mechanism underlying hair follicle development and wool-related traits in Merino sheep by combining transcriptome and DNA methylome approaches [32], and by mining key genes that regulate Merino coarse and fine wool through m6A methylomics [7]. However, few studies have used whole-transcriptome m6A methylation analyses to clarify the regulation of wool fiber diameter. As a common modification type in eukaryotic organisms, m6A methylation plays an important role in gene expression [33]; therefore, analyzing the role of m6A methylation in the regulation of wool fiber diameter can improve our understanding of the regulation of wool fineness. In this study, skin samples with different wool fiber diameter ranges were selected, covering the wool diameters required for high-end wool products and low-end wear-resistant and dirt-resistant wool products. By setting different controls, the effect of m6A methylation with respect to wool fineness was revealed.

Based on the analysis of identified m6A peaks and gene analysis, the SF group and M group showed the greatest quantitative difference, while these two groups also exhibited the most significant phenotypic differences. This seems to indicate that the degree of m6A methylation modification correlates with phenotypic differences. Hua et al [7] studied wool fineness in cashmere goats; however, the total number of peaks identified was significantly lower than the number of peaks identified in this study. It is possible that m6A methylation is relatively conserved, and the degree of m6A methylation modification differs among species. In the EF/SF, M/SF, and M/EF groups, most of the differential m6A peaks were located in exons, consistent with general findings for m6A methylation [34]. The proportion of hypermethylation was extremely high in the M/SF and M/EF groups. We excavated genes that were hypermethylated in the three comparisons, including *BACH2* [35], *FHOD1* [36], *NCKAP5* [37], *RASSF7* [38], *SEA6B* [39]. Most of these genes are disease markers or contribute to immune processes. An enrichment analysis showed that these genes are involved in cell component-related pathways, explaining why they are differentially methylated across the three comparisons. We found that some DMGs in the EF/SF and M/SF groups were enriched in the positive regulation of transcription of Notch receptor target, Wnt signaling pathway, negative regulation of Notch signaling pathway, regulation of transforming growth factor beta receptor signaling pathway, and other related pathways. These pathways are involved in embryonic development and the maintenance of hair follicles, promoting the interaction between different cell types in hair follicles, regulating the hair follicle cycle, and affecting the dynamics of the hair growth cycle [25,26,40,41]. The M/SF group, in particular, was enriched in a number of key pathways. Most of the DMGs in these pathways were hypermethylated and had one m6 peak. Surprisingly, we also found two genes, RNF43 and SMAD3, with known roles in hair structure and fineness [42,43]. These two genes were hypermethylated and contained two m6A peaks in the comparisons between the M and SF groups. Therefore, we speculate that the phenotypic differences between the M and SF groups may be caused by hypermethylation. This is consistent with previous results indicating that the methylation in the M group is higher than that in the SF group. The EF/SF group was also enriched in the Notch-related pathway, and the number of hypermethylated DMGs related to this pathway was consistent with the number of hypomethylated DMGs; this may be due to the relatively small difference in wool fiber diameter between the EF and SF groups. Secondly, the EF/SF group was highly enriched in energy metabolism and intercellular signal transduction, indicating that the mechanisms determining differences in wool fineness between the EF and SF groups was particularly complicated.

The combined m6A methylation and transcriptome analysis showed that the degree of m6A methylation was positively correlated with gene expression, consistent with the findings of Xi et al., who found that highly methylated mRNA often had higher translation efficiencies [44]. *YTHDF1* recognizes and binds m6A-modified mRNAs during methylation, prompting their translation, while *YTHDF2* degrades mRNAs [45,46]. *YTHDF1* was up-regulated and *YTHDF2* was down-regulated in the M/EF and M/SF groups, and both *YTHDF1* and *YTHDF2* were down-regulated in the EF/SF group. The expression patterns of *YTHDF1* and *YTHDF2* may be responsible for the presence of a large number of genes that were hypermethylated and significantly up-regulated in the M/EF and M/SF groups, whereas relatively few similar genes were found in the EF/SF group. By analyzing the functional pathways of genes in the EF/SF group, FOS and *CACNA1E*, both DMGs and DEGs, were enriched in the MAPK pathway, which is involved in the hair follicle cycle and may regulate the growth of hair follicle stem cells [47,48]. Both genes were hypomethylated in the EF/SF group, and the rest of the hypomethylated genes were enriched in the pathway related to biological clock synchronization, which mainly regulates physiological rhythms and contributes to adaptability. Pathways related to hypermethylated genes were generally related to diseases; we hypothesized that the phenotypic differences in the EF/SF group may result from hypomethylation, which alters the expression of genes in these pathways. However, this remains to be verified. As noted, the phenotypic differences between the M and SF groups may be caused by hypermethylation. We found that *CAMK2B* was hypermethylated and significantly up-regulated; these gene is a downstream target gene of p38α MAPK [49] a member of the MAPK family. It has been reported that p38 MAPK negatively regulates epidermal growth factor by inhibiting extracellular signal-regulated kinases in human skin fibroblasts, which ultimately affects skin development. p38α MAPK, a p38 MAPK isoform, plays a major role in this process [50]. It is hypothesized that *CAMK2B* is involved in skin development via the downstream target gene *p38αMAPK*, thereby affecting the development of hair follicles. Methylation is involved in the whole process by changing the expression of *CAMK2B*.

lncRNAs and circRNAs, crucial for regulating biological processes, are common m6A modification targets. Recent reports have confirmed that lncRNAs play a crucial role in regulating the proliferation and differentiation of hair follicle stem cells through the TGF-β1-mediated Wnt/β-catenin pathway [51]. Additionally, TCONS _ 00279168 has been identified as a key regulator of ATP1B4 and FGF12 in sheep hair follicles [52]. Hui et al [10] found significantly higher levels of m6A-modified circRNAs, such as m6A-circRNA-ZNF638, -TULP4, and -DNAJB6, in hair follicles during the anagen phase of the skin than during other periods and emphasized that these m6A-modified circRNAs may influence hair follicle development. In this study, by mining lncRNAs and circRNAs in skin tissues, we found that most enriched pathways for lncRNAs in the three groups did not have a direct relationship with wool fiber diameter, and the NK-kappaB and TGF-β pathways were present only in the EF/SF and M/EF groups. Surprisingly, lncRNAs in these pathways were not modified by m6A methylation. we analyzed the degree of m6A methylation of circRNAs in pathways associated with wool fiber diameter and found that circRNAs were methylated in all three comparisons, with higher circRNA methylation in the EF group than in the SF group and higher circRNA methylation in the M group than in the SF group, again suggesting that hypermethylation affects wool fiber diameter. We constructed a relevant network diagram, and circRNAs were a hotspot in the regulatory network. NF-kappaB and Notch were the main pathways related to circRNAs, and these were also the main pathways related to DMGs, suggesting that they have important roles in the regulation of wool fiber diameter. lncRNAs were also involved in the newly constructed regulatory network; however, there is still no clear explanation for the lack of methylation of lncRNAs. It is likely that methylated lncRNAs are more susceptible to degradation. Further studies are needed to confirm this hypothesis.

## CONCLUSION

In this study, we united m6A methylomics and transcriptomics to unearth a large number of meaningful methylation-modified RNAs, revealed the effect of hypermethylation on wool fiber diameter, and obtained candidate biological pathways involved in the regulatory effects of methylation. The results of the present study emphasize the importance of methylation in the regulation of wool fiber diameter, further improve our understanding of the genetic mechanism underlying this trait, and provide a theoretical basis for the optimization of wool fiber.

## SUPPLEMENTARY MATERIAL

**Supplement 1**. M6A methylation peak distribution.

**Supplement 2**. Data distribution of circRNA in skin tissue.

**Supplement 3**. Total lncRNAs data.

**Supplement 4**. lncRNA and cirRNA difference Peak KEGG analysis.
