# Supplementary material for: The mechanism of m6A methylation analysis of the transcriptome to regulate the diameter of Alpine Merino wool fiber
Source: Anim Biosci. 2025 Aug 25;39(2):250347. doi: 10.5713/ab.25.0347 (PMC12877394; doi:10.5713/ab.25.0347)
Supplement: Supplementary file 4 [file ab-25-0347-Supplementary-4.pdf]

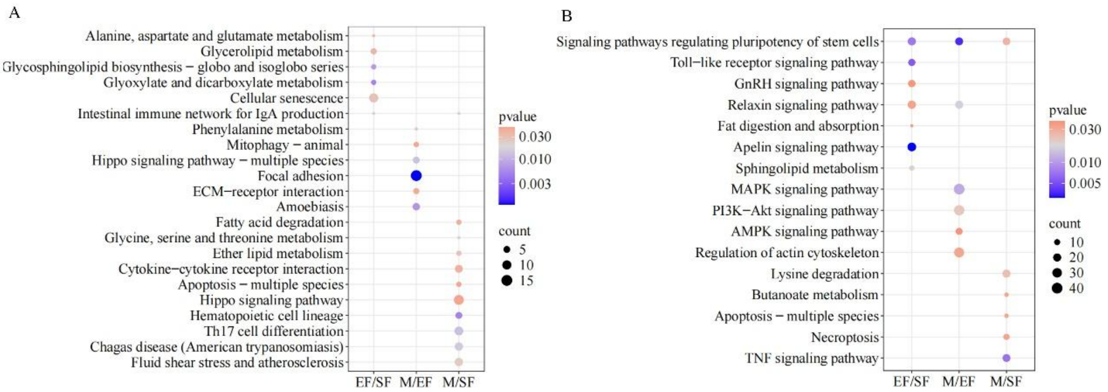

FigS1 lncRNA and cirRNA difference Peak KEGG analysis. A: lncRNA differential Peak KEGG analysis. B: cirRNA differential Peak KEGG analysis.
